# Evidence for decreased parasympathetic response to a novel peer interaction in older children with autism spectrum disorder: a case-control study

**DOI:** 10.1186/s11689-020-09354-x

**Published:** 2021-01-09

**Authors:** Rachael A. Muscatello, Simon N. Vandekar, Blythe A. Corbett

**Affiliations:** 1 Neuroscience Graduate Program, Vanderbilt University, Village at Vanderbilt, Suite 2200, 1500 21st Avenue South, Nashville, TN 37212 USA; 2 Department of Psychiatry and Behavioral Sciences, Vanderbilt University Medical Center, PMB 40, 230 Appleton Place, Nashville, TN 37203 USA; 3 Department of Biostatistics, Vanderbilt University Medical Center, 2525 West End, Suite 1130, Office 1136, Nashville, TN 37203 USA; 4 Department of Psychology, Vanderbilt University, PMB 40, 230 Appleton Place, Nashville, TN 37203 USA; 5 Vanderbilt Kennedy Center, Vanderbilt University Medical Center, PMB 40, 230 Appleton Place, Nashville, TN 37203 USA

**Keywords:** Autism spectrum disorder, Autonomic nervous system, Respiratory sinus arrhythmia, Pre-ejection period, Social, Age, Stress

## Abstract

**Background:**

Individuals with autism spectrum disorder (ASD) often experience elevated stress during social interactions and may have difficulty forming and maintaining peer relationships. The autonomic nervous system (ANS) directs physiological changes in the body in response to a number of environmental stimuli, including social encounters. Evidence suggests the flexibility of the ANS response is an important driving factor in shaping social behavior. For youth with ASD, increased stress response and/or atypical ANS regulation to benign social encounters may therefore influence social behaviors, and, along with developmental and experiential factors, shape psychological outcomes.

**Methods:**

The current study measured ANS response to a peer-based social interaction paradigm in 50 typically developing (TD) children and 50 children with ASD (ages 10–13). Respiratory sinus arrhythmia (RSA), a cardiac measure of parasympathetic influence on the heart, and pre-ejection period (PEP), a sympathetic indicator, were collected. Participants engaged in a friendly, face-to-face conversation with a novel, same-aged peer, and physiological data were collected continuously before and during the interaction. Participants also reported on state anxiety following the interaction, while parents reported on the child’s social functioning and number of social difficulties.

**Results:**

Linear mixed models revealed that, while there were no diagnostic effects for RSA or PEP, older youth with ASD appeared to demonstrate a blunted parasympathetic (RSA) response. Further, increased severity of parent-reported social symptoms was associated with lower RSA. Youth with ASD reported more anxiety following the interaction; however, symptoms were not related to RSA or PEP response based on linear mixed modeling.

**Conclusions:**

Physiological regulation, age, and social functioning likely influence stress responses to peer interactions for youth with ASD. Parasympathetic functioning, as opposed to sympathetic arousal, may be especially important in behavioral regulation, as older youth with ASD demonstrated atypical regulation and response to the social interaction paradigm. Future studies should help to further elucidate the developmental factors contributing to stress responses in ASD, the impact of physiological response on observable social behavior, and potential long-term consequences of chronic social stress in youth with ASD.

**Supplementary Information:**

The online version contains supplementary material available at 10.1186/s11689-020-09354-x.

## Background

Autism spectrum disorder (ASD) is a neurodevelopmental disorder now estimated to affect 1 in 54 children in the USA [1]. Symptoms of ASD are defined across two core diagnostic domains—impairments in social interaction and communication, and restrictive and repetitive patterns and behaviors [2]. Thus, individuals often have significant difficulty engaging with others, responding to novel social situations, and often find peer interactions to be stressful [3–6]. Nevertheless, humans are inherently social creatures, and despite social challenges, children must interact with peers nearly every day—in the classroom, on the playground, and in the community.

The autonomic nervous system (ANS) is separated into two branches with primarily opposing functions, the parasympathetic (PNS) and sympathetic nervous systems (SNS). The PNS is described as the “rest and digest” branch by conserving energy as it slows heart rate (bradycardia), lowers blood pressure, decreases respiration, and increases intestinal activity, among other regulatory actions [7]. In contrast, the metabolically demanding SNS supports “fight or flight” responses for mobilization to threat, including, but not limited to, increased heart rate and respiration. The sinoatrial (SA) node, or pacemaker, of the heart is dually innervated by the PNS and SNS [8]. Non-invasive measures of cardiac function can identify the individual contributions of each branch, serving as useful markers of change in PNS and SNS activity (e.g., )[9, 10]. For example, changes in beat-to-beat heart rate (heart rate variability; HRV) in conjunction with high-frequency range respiration, or respiratory sinus arrhythmia (RSA), indexes PNS influences. Further, the pre-ejection period (PEP), derived by impedance cardiography to detect volumetric changes, is a metric of time from electrical stimulation to mechanical opening of the aortic value and is a validated measure of pure SNS function (e.g. )[10].

The ANS includes a neural network in which efferent signals originating from medullary brainstem regions [11, 12] affect functioning of peripheral visceral organs, including the heart. The dually innervated SA node is said to be under tonic parasympathetic inhibition via the myelinated vagal nerve [11, 13]. This “vagal brake” regulates behavior through maintenance and balance of PNS influence to the heart, thus allowing for changes in heart rate as parasympathetic regulation changes in response to changing environmental stimuli [11, 13, 14]. Therefore, in the presence of a stressor, removal of the “vagal brake” can allow for increases in heart rate and respiration without engaging the metabolically demanding SNS [15].

Vagal flexibility [16] is believed to play an important role in determining social behavior. According to the Polyvagal Theory [11], the parasympathetically-mediated Social Engagement System [13, 14, 17] is active during calm visceral states, thus promoting activation of the interconnected craniofacial nerves and their associated motor behaviors. These somatomotor components of the system control a number of actions relevant for social behavior, including but not limited to, eye movement (eye contact), vocalization (language), and head turning (social orienting) [18]. For example, individuals who demonstrate more cooperativity and sociability tend to have higher PNS regulation [19–21]. Additionally, young adults with higher vagal tone are more socially engaged than their peers with lower PNS regulation [22]. However, in cases of more severe threat, the SNS will activate, presumably inhibiting parasympathetic systems and blocking the Social Engagement System while initiating the fight or flight response to the stressor.

It has been noted that many of the behaviors associated with the Social Engagement System, including eye gaze, language and vocalization production, and emotional expression [13, 14, 17], are often impaired in a number of neurological conditions, most notably, autism spectrum disorder [18, 23]. The autonomic system may function atypically in ASD, evidenced by reductions in resting PNS regulation relative to TD peers [24–26]. Several studies additionally cite atypical PNS and SNS reactivity in response to stress (e.g.), [27–30]. In a study of school-aged children, those with ASD demonstrated lower RSA during interactions with unfamiliar peers; moreover, the reduction in RSA was associated with more social problems and problem behaviors [26]. A similar reduction in parasympathetic regulation, along with sympathetic hyperarousal, was seen in ASD children, compared to TD controls, when interacting with a familiar partner [30]. In the context of social play, higher resting PNS regulation has been associated with more gestures and sharing behavior in young children with ASD during play with an adult actor [31]. Therefore, social difficulties in ASD may be, in part, explained by failures of the parasympathetically-mediated vagal nerve to efficiently regulate the *Social Engagement System*, where PNS withdrawal and/or SNS hyperarousal inhibits the facial nerves and associated motor neurons responsible for many social behaviors [18].

Stress reactivity is dynamic, with physiological responsivity influenced by a number of factors including age or development (e.g., [32]) and social variables (i.e., peer support) (e.g.), [33]. During a naturalistic play protocol, the Peer Interaction Paradigm (PIP) (3), many youth with ASD demonstrate elevated stress reactivity of a neuroendocrine system, the hypothalamic-pituitary-adrenal (HPA) axis, relative to TD peers. Moreover, these effects are further modified by age. In two studies of 8 to 12 year olds with ASD or TD, older youth with ASD showed a greater stress response to the social interaction from baseline relative to their typically developing (TD) peers and younger youth with ASD [3], suggesting the age effects may be related to other social factors such as increased insight and more exposure to negative social experiences in the older youth. Similar developmental patterns have been noted in the autonomic system, where school-aged TD children exhibited differing RSA suppression responses to stress according to their age [34]. Specifically, younger children (8–11 years) demonstrated greater suppression to a cognitive stressor compared to older youth (12–15 years). However, no differences were noted between age groups in the social task, involving hearing an argument [34]. Collectively, these findings provide evidence that age may influence physiological stress responses, with further research needed to elucidate these possible relationships between autonomic stress reactivity, social functioning, and development in youth with ASD.

The current study sought to extend previous studies by examining physiological stress response to a friendly social encounter (TSST-F) in youth with and without ASD. Specifically, we measured PNS and SNS responses over time—from baseline, throughout the interaction, until recovery—to examine whether youth with ASD showed a unique pattern of response relative to TD peers. Given previous research in similar physiological systems (HPA axis) using peer interaction paradigms (e.g.), [6], as well as noted developmental effects on ANS regulation and responsivity (e.g.), [35, 36], we hypothesized youth with ASD, especially older children, would show heightened stress and arousal to the current social interaction paradigm. Additionally, we expected findings would be consistent with the Polyvagal Theory and Social Engagement System (e.g.), [14], such that social difficulties in ASD would be associated with a dysregulated physiological state. Specifically, we predicted that children with ASD would show: (1a) autonomic hyperarousal demonstrated by lower PNS and elevated SNS, representing a chronic mobilization state, (1b) atypical autonomic flexibility in response to the social interaction with less change in PNS response and heightened SNS reactivity; (2) stress response across the interaction would be modified by age, with older age associated with elevated physiological arousal; and (3) ANS hyperarousal would be associated with more severe social symptoms and state anxiety.

## Methods

### Participants

Participants included 100 children 10 to 13 years of age, with ASD (*n* = 50, mean age = 11.48) or typical development (*n* = 50, mean age = 11.35). Gender was matched between groups, with 14 females in each group. As part of a longitudinal study of pubertal development [37], families were enrolled from the community within a 200-mile radius through research registries, university-wide announcements, autism- and child-development clinics, and social media. The sample consisted of 85.0% Caucasian, 6.0% Black/African American, 1.0% Asian, and 8.0% Mixed Race. Moreover, 7.0% of the sample was Hispanic. Parental education served as a proxy for socioeconomic status; 50% of parents had a bachelor’s or master’s, 30% associate’s or high school, and 20% doctorate or professional. An estimated 42% of children with ASD have been reported to take at least one psychotropic medication [38]; thus, study criteria did not require participants to be medication-naïve in order to be more representative of the overall ASD population. However, participants prescribed medications that may directly affect the ANS (e.g., stimulants) [39] were not enrolled. In total, 18 children with ASD were on medications at the time of the study, including primarily antihistamines, melatonin, or selective-serotonin reuptake inhibitors (SSRIs). Three TD participants were taking over-the-counter antihistamines at the time of enrollment for management of seasonal allergies.

### Diagnostic criteria

All participants were required to have an estimated IQ ≥ 70, as measured by the Wechsler Scale of Abbreviate Intelligence (WASI-II), [40] in order to ensure adequate language to meet the social demands of the interaction task and to ensure the ability to complete self-report forms associated with the larger longitudinal study [37]. Parents completed the Social Communication Questionnaire-Lifetime (SCQ-L) [41], a screening questionnaire for identifying symptoms of ASD showing good sensitivity (0.88) and specificity (0.72) [42]. In order to be included in the study, TD youth required a score < 10 on the SCQ-L based on parent-report. Additionally, TD participants could not have a biological sibling with ASD. Diagnosis of ASD was based on DSM-5 criteria [2] and established by (1) previous diagnosis by psychiatrist, psychologist, or clinician with autism expertise; (2) current clinical judgment; and (3) corroborated by the Autism Diagnostic Observation Schedule, 2^nd^ edition [43], administered by research-reliable personnel.

### Procedures

The study was carried out in accordance with the Code of Ethics of the World Medical Association (Declaration of Helsinki). The Vanderbilt Institutional Review Board approved all study procedures. In compliance with the Institutional Review Board, informed written consent and verbal assent was obtained from all parent/guardians and children, respectively, prior to inclusion in the study. The study was completed across two visits to a university research lab. Diagnostic and cognitive measures were administered at visit 1. Parents also completed the Child Behavior Checklist (CBCL) [44] and Social Responsiveness Scale (SRS-2) [45]. At visit 2, participants were exposed to the social interaction protocol, the Trier Social Stress Test-Friendly (TSST-F) [46], and completed all physiological data collection

### Trier Social Stress Test-Friendly

**Fig. 1:**
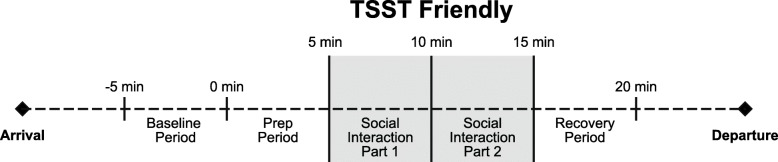
Trier Social Stress Test-Friendly. Timeline schematic for 20-min TSST-F social interaction paradigm

The Trier Social Stress Test-Friendly [46] is an alternative form of the original TSST [47], which has been shown to elicit a physiological stress response from social evaluative threat. The TSST-F, however, consists of a more “friendly” protocol, in which participants describe him or herself and/or a favorite book, movie, hobbies, or other interest in front of a novel peer of the same sex who shows encouragement (smiles, nods, shows interest, maintains eye contact) and asks follow-up questions. The “friendly” TSST, unlike the original TSST, produces no physiological stress response in typically developing individuals [46, 48] and parallels other peer interaction paradigms [3]. After a 5-min resting baseline period when participants were asked to sit quietly, the instructions were read aloud and youth given the opportunity to prepare what they would like to share during a 5-min preparation period. During this preparation period, research personnel are not to engage with the participant, and if the child asks questions, personnel simply repeated the instructions, that they are “to prepare what they would like to say to the other child.” Following the prep period was the 10-min social interaction with the novel peer. Lastly, a recovery period measured return to baseline, and participants were again asked to sit quietly and calmly for 5 minutes. Physiological data was collected continuously throughout the paradigm, including at preparation, through the social interaction (divided into two, 5-min segments—part 1 and part 2), and recovery (see Fig. 1). The 20-min TSST-F paradigm requires reciprocal social interaction with a novel trained peer, conceptualized to be a more potent stressor for children with ASD. Peers were thoroughly trained using a manualized protocol, review of videotaped TSST-F sessions, and practice with senior research personnel prior to working with a participant. Furthermore, the peers were monitored to maintain consistent implementation of the protocols. Each administration of the TSST-F was recorded for behavioral coding purposes, and videos were routinely checked to ensure peers maintained social interest without talking for > 50% of the conversation. If deviations in the protocol were noted, booster training sessions were promptly provided.

### Dependent measures

#### Social symptoms and perceived anxiety

The *Child Behavior Checklist* (*CBCL*) [44] is a parent-report measure of behavioral and emotional problems in children ages 6–18 years. Scores are rated on a Likert scale from 0 (“not true”) to 2 (“very often true”). The CBCL has demonstrated good-to-excellent reliability in ASD, with individual scale reliabilities ranging from 0.69 to 0.94, including a reliability of 0.84 for the Social Problems domain [49]. Due to the a priori hypotheses regarding social symptoms and physiology, we specifically examined the social problems subscale. Previously, youth with ASD demonstrated significantly elevated scores on the social problems subscale relative to controls [50]. Raw scores were used in analyses, as recommended in the CBCL manual [44].

The *State-Trait Anxiety Inventory for Children* (*STAIC*) [51] is a self-report measure of anxiety, completed by participants, in which an individual describes how he/she is currently feeling (state) and how he/she usually feels (trait). Previous studies have found youth with ASD are able to identify anxiety following stressors [52–54], including reporting elevated state anxiety following a social interaction [55].

The *Social Responsiveness Scale* (*SRS-2*) [45] is a parent-report questionnaire developed to identify the severity of ASD symptoms across several domains. Domain and total scores are presented as standardized *T* scores. The SRS shows high sensitivities (0.74 to 0.80) and specificities (0.69 to 1.00) for ASD [56]. Analyses included SRS total scores in order to examine total range of ASD-related symptoms.

#### Heart rate variability

Cardiac autonomic measures were collected using MindWare Mobile Impedance Cardiograph units (MindWare Technologies LTD, Gahanna, OH) for synchronized electrocardiography (ECG) and respiration data collection using a seven-electrode configuration. Participants were told they would be wearing “stickers” throughout the protocol, and a color cartoon was provided to illustrate the location of the electrodes. Participants were given the opportunity to place an electrode on their hand prior to placement, and a five-minute acclimation period followed electrode placement to allow children time to become comfortable with the sensory aspects of the protocol. All 100 participants agreed to complete the heart rate collection and were able to comfortably tolerate the electrode placement.

Resting ANS regulation was acquired using a 5-min baseline collection period in which participants were instructed to sit quietly without engaging in other tasks. During the social interaction, cardiac measures were collected continuously, calculated on a minute-by-minute basis, and averaged into 5-min epochs for each major period of the paradigm—baseline, prep, social interaction (two, 5-min segments—parts 1 and 2), and recovery (see Fig. 1).

Parasympathetic regulation was indexed using respiratory sinus arrhythmia (RSA) and derived in accordance with the guidelines set forth by the Society for Psychophysiological Research committee on heart rate variability [9, 57]. ECG signal was sampled at 500 Hz and analyzed using the Heart Rate Variability Software Suite provided by MindWare Technologies (MindWare Technologies LTD, Gahanna, OH). RSA was quantified as the integral power within the respiratory frequency band (0.12 to 0.40 Hz), and respiration was monitored by impedance cardiography [58]. The respiration signal was displayed to ensure that the values were within the designated frequency band. Respiratory frequency was confirmed to lie within the high frequency/RSA band (0.12–0.40 Hz) for all participants. Of the total collected data, 1.0% were excluded due to excessive motion artifact or cardiac arrhythmias. RSA was measured in ln (ms^2^).

Pre-ejection period (PEP) was collected using impedance cardiography and represents the interval from electrical stimulation to the mechanical opening of the aorta. PEP was processed with MindWare Technologies Impedance Cardiography Analysis Software (MindWare Technologies, LTD, Gahanna, OH) and calculated as the distance (in ms) from the ECG Q-point of the QRS complex to the B point of the impedance waveform, which corresponds with the time from ventricular depolarization to aortic valve opening [10]. PEP was ensemble-averaged for each one-minute epoch by the MindWare software, and B-point was calculated at 55% of the R-Z interval (time to dZ/dt peak) [59]. The QRS complex and dZ/dt signal were confirmed by visual inspection (RAM). Due to equipment malfunction or excessive artifact in the impedance signal, 14 participants had incomplete PEP data (TD, *n* = 6, ASD, *n* = 8,χ^2^(1) = 0.33, *p* = 0.56). An additional 2.0% of total data was excluded due to values less than 70 ms, which falls below physiological norms [60] and is suggestive of equipment or measurement error.

### Statistical analysis

Demographic, diagnostic, and inclusion variables were compared between ASD and TD groups using independent sample *t* tests. The Welch degree of freedom approximation was used to correct for violations of homogeneity of variance. RSA and PEP values were normally distributed and free of extreme outliers.

Hypotheses were tested using linear mixed models. Time was modeled continuously with linear and quadratic terms, calculated from five time points—baseline, prep, social interaction part 1, social interaction part 2, and recovery—with baseline centered as time zero. To examine whether ASD diagnosis was associated with autonomic hyperarousal, we tested the main effect of diagnosis, followed by the diagnosis*time interaction to determine if change in RSA or PEP from baseline differed by diagnosis. Additionally, we tested the main effect of age to examine the hypothesis that older age would be associated with increased stress (lower RSA and PEP). Subsequently, an age*diagnosis interaction tested whether diagnostic groups showed differences in age effects on RSA/PEP. Finally, exploratory models were investigated with CBCL social problems, SRS total problems, and STAIC state anxiety as potential covariates or effect modifiers. All statistical analyses were conducted using IBM SPSS Version 26 [61].

## Results

### Demographics

Age did not differ between ASD and TD groups (see Table 1). While there was a significant difference based on IQ, the ASD group was well within the average range of functioning. As both groups fell within the average range for IQ, we did not expect significant effects of IQ on autonomic response. Nevertheless, all models were also run while controlling for IQ, and the results were largely unchanged with no differences in the significance level of the findings (see Supplemental Tables). Children with ASD were rated by their parents as having significantly more social symptoms on the CBCL and SRS. The ASD group also self-reported greater anxiety after the TSST-F relative to TD youth. Within the ASD group, medication status (taking medication vs. no medications) was not associated with any of the demographic or outcome variables.

**Table 1:** Demographic and dependent variables

	ASD			TD			*t*	df	*p*
	M	SD	Range	M	SD	Range			
Age	11.48	1.06	10.0–13.7	11.35	1.05	10.0–13.9	− 0.62	97.99	0.54
IQ**	100.28	17.83	71–129	120.20	13.33	88–145	6.33	90.74	< 0.001
ADOS	12.69	4.73	7–22	–	–	–	–	–	–
SCQ**	17.70	8.08	7–33	2.24	2.20	0–8	− 12.88	56.26	< 0.001
CBCL social problems (raw)**	7.20	4.23	0–18	1.88	2.47	0–10	− 7.68	78.98	< 0.001
STAIC (*state*)*	31.18	7.15	23–51	28.62	4.73	20–42	− 2.11	85.04	0.04
**SRS total (*T*** score)**	68.46	10.40	47–90	47.08	6.21	37–60	− 12.48	80.01	< 0.001

*IQ* intelligence quotient, *ADOS* Autism Diagnostic Observation Schedule, *CBCL* Child Behavior Checklist, *SCQ* Social Communication Questionnaire, *STAIC* State-Trait Anxiety Inventory for Children, *SRS* Social Responsiveness Scale, *ASD* autism spectrum disorder, *TD* typically developing

*p < 0.05

**p < 0.001

### RSA regulation and responsivity

**Fig. 2:**
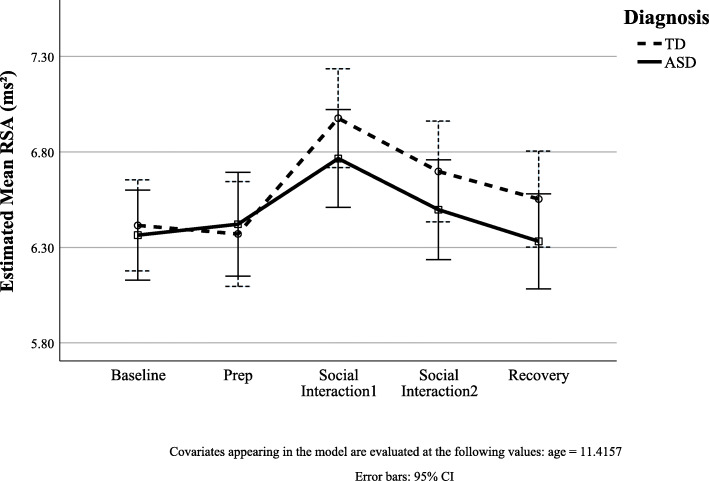
Estimated RSA during TSST-F by diagnosis. ASD and TD youth did not differ in mean RSA (intercept) or RSA responsivity (slope) during the TSST-F paradigm while controlling for age

The initial model with diagnosis, time, and nonlinear age to model RSA was significantly improved relative to a trivial model with constant RSA level (χ^2^(4) = 46.55, *p* < 0.001). Wald tests using type 3 sum of squares showed little evidence for a main effect of diagnosis (F(1,99) = 0.39, *p* = 0.53). Further, addition of the diagnosis*time interactions to the model was not significant (χ^2^(2) = 4.41, *p* = 0.11; see Table 2 for parameter estimates), suggesting the rate of change in RSA with respect to time did not differ in the ASD group relative to the TDs (Fig. 2).

**Table 2:** Model estimates of physiological variables change with a diagnosis by time interaction

Variable	Estimate	SE	df	***t***	***p***	(95% CI)
RSA						
Intercept	5.82	0.89	99.61	6.57	< 0.001	(4.06, 7.58)
Diagnosis	0.02	0.18	139.96	0.14	0.89	(− 0.32, 0.38)
Time	0.37	0.07	394.14	5.54	< 0.001	(0.24, 0.51)
Time^2^	− 0.08	0.02	394.09	− 4.89	< 0.001	(− 0.11, − 0.05)
Age	0.04	0.08	98.95	0.56	0.57	(− 0.11, 0.20)
Diagnosis*time	− 0.08	0.10	394.09	− 0.87	0.39	(− 0.27, 0.10)
Diagnosis*time^2^	0.01	0.02	394.06	0.29	0.77	(− 0.04, 0.05)
PEP						
Intercept	70.06	11.01	90.34	6.36	< 0.001	(48.19, 91.93)
Diagnosis	1.72	2.08	104.69	0.83	0.41	(− 2.41, 5.85)
Time	0.34	0.54	340.01	0.64	0.52	(− 0.71, 1.40)
Time^2^	− 0.10	0.13	339.44	− 0.82	0.41	(− 0.36, 0.15)
Age	1.66	0.96	90.12	1.73	0.09	(− 0.25, 3.57)
Diagnosis*time	0.18	0.77	339.72	0.23	0.81	(− 1.34, 1.70)
Diagnosis*time^2^	− 0.10	0.18	339.31	− 0.55	0.58	(− 0.46, 0.26)

**Fig. 3:**
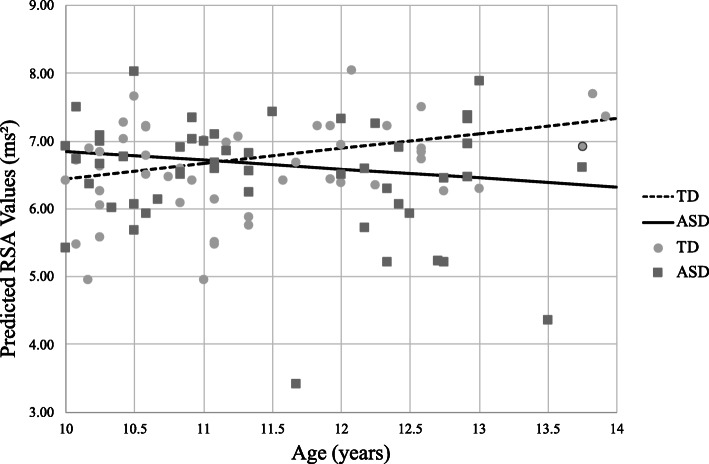
Predicted RSA values by age differ in ASD and TD youth. The figure represents estimated RSA for children ages 10–13 years while controlling for time. Youth with ASD (solid line) demonstrate a slower rate of change in RSA compared to TD youth (dashed line). Markers represent average RSA of the entire task, and slopes (lines) represent projected linear change in RSA by age as estimated from linear mixed model

A second model including diagnosis, time, age, and an interaction term for diagnosis by age showed a main effect for age (*p* = 0.04), indicating increased age is associated with higher RSA in the TD group. Further, there was a significant diagnosis*age interaction (*p* = 0.02; see Table 3 for model parameter estimates); thus, the rate of change in RSA with respect to age is slower in ASD relative to TD (Fig. 3). The effect of age on RSA for the ASD group was equal to a change of − 0.13 ms^2^ per year, while in the TD group the RSA change equaled 0.22 ms^2^ per year.

**Table 3:** Model estimates of physiological variable change including the age by diagnosis interaction

Variable	Estimate	SE	df	***t***	***p***	(95% CI)
RSA						
Intercept	3.86	1.22	99.12	3.16	0.002	(1.44, 6.28)
Diagnosis	3.92	1.72	98.95	2.28	0.02	(0.50, 7.35)
Time	0.34	0.05	394.09	6.97	< 0.001	(0.24, 0.43)
Time^2^	− 0.08	0.01	394.06	− 6.63	< 0.001	(− 0.10, − 0.05)
Age	0.22	0.11	98.95	2.08	0.04	(0.01, 0.43)
Diagnosis*age	− 0.35	0.15	98.95	− 2.34	0.02	(− 0.65, − 0.05)
PEP						
Intercept	62.26	15.00	89.97	4.15	< 0.001	(32.47, 92.06)
Diagnosis	18.37	22.02	90.14	0.83	0.41	(− 25.38, 62.13)
Time	0.43	0.39	339.78	1.11	0.27	(− 0.33, 1.19)
Time^2^	− 0.15	0.09	339.36	− 1.68	0.09	(− 0.34, 0.03)
Age	2.36	1.31	89.89	1.79	0.08	(− 0.25, 4.97)
Diagnosis*age	− 1.48	1.92	90.18	− 0.77	0.44	(− 5.29, 2.33)

**Fig. 4:**
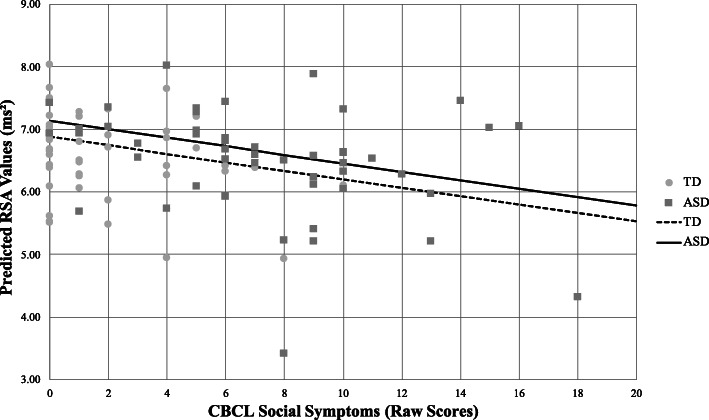
Predicted RSA by social symptom severity. The figure represents predicted RSA according to a number of reported social problems on the CBCL while controlling for age and time. Both groups demonstrate a negative association between RSA and social symptoms (solid and dashed lines), such that the lowest RSA was associated with more severe social problems. Markers represent average RSA of the entire task, and slopes (lines) represent projected linear change in RSA by social symptom severity as estimated from linear mixed model

Exploratory models adding the social problems domain of the CBCL to the base model with diagnosis, age, and time significantly improved fit (χ^2^(1) = 8.63, *p* = 0.003); however, CBCL social problems*time effects were not significant predictors of RSA (χ^2^(2) = 3.61, *p* = 0.16). Thus, mean RSA differed based on the severity of scores on the social problems domain (Fig. 4), but the change in RSA over time (slope) did not. Models for STAIC state anxiety (χ^2^(1) = 0.84, *p* = 0.36) or SRS total score (χ^2^(1) = 1.184, *p* = 0.28) were not a significant improvement over the base model with diagnosis, time, and age.

Finally, exploratory ad hoc models investigated possible three-way interactions between diagnosis, age, and time. There was not sufficient evidence for a significant three-way, nonlinear interaction for diagnosis, age, and time (χ^2^(2) = 4.26, *p* = 0.12); however, the current sample may have been underpowered to test these higher-order interactions. Therefore, an estimate of effect size was calculated using a recently proposed effect size index (*S* [62];). This index is equal to ½ Cohen’s d. The effect size for the interaction was *S* = 0.160, which falls in Cohen’s small to medium effect range [63].

### PEP regulation and responsivity

The hypothesized model of diagnosis, time, and age was significant relative to a trivial model with constant PEP (χ^2^(4) = 9.501, *p* = 0.05). The addition of diagnosis*time interaction terms were not significant (χ^2^(2) = 1.345, *p* = 0.51). A second model including an interaction term for diagnosis by age was not significant (χ^2^(1) = 0.591, *p* = 0.44). See Tables 2 and 3 for detailed model results. Further models with social symptoms and anxiety were non-significant in predicting PEP (all *p* > 0.05).

## Discussion

The primary objective of the current study was to determine whether youth with ASD showed differential physiological responses to a naturalistic social interaction task. Results revealed a profile of stress and arousal in youth with ASD in which physiological system, age, and social symptoms may all influence peer interactions. The PNS appeared to be sensitive to developmental effects, with older ASD youth evidencing lower RSA. In contrast, we did not find evidence that the SNS was sensitive to the TSST-F or differences associated with ASD symptoms. Youth with ASD may be in a state of autonomic hyperarousal from PNS withdrawal, which may not only influence social behavior, but can also increase risk for stress-related conditions (e.g., [64–68]), further emphasizing the important implications for clearly defining ANS functioning in ASD youth.

RSA and PEP stress response across the paradigm did not differ between youth with ASD and TD. In regard to PEP, the SNS is often considered a second line of defense, only activated during more severe conditions of stress [15]. The PNS is more flexible, facilitating autonomic responses to dynamic conditions via changes in vagal tone, or suppression and activation of the “vagal brake” (e.g.), [14]. Therefore, the PNS as measured by RSA would be expected to change in response to a wider variety of stimuli. The lack of diagnostic effects on RSA regulation and response did not support our hypotheses and conflict with previous studies of similar-aged youth with ASD. For example, Van Hecke and colleagues (2009) reported that 8–12-year-old youth with ASD demonstrated lower RSA overall, and in particular, a decrease in RSA to a video of an unfamiliar adult. Similarly, a relatively small sample of school-aged youth with ASD was reported to show reduced RSA across baseline, cognitive, and social tasks [69]. However, others have found no differences in heart rate variability within a similar age range, while also noting that age had a significant effect on many physiological stress variables [70]. It may be important to consider age when examining stress responses, as it has been posited that children of certain ages may find certain tasks more or less stressful [34].

**Fig. 5:**
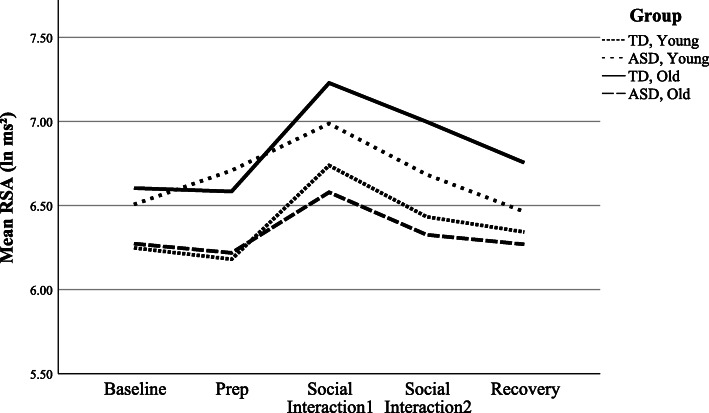
Projected RSA response profiles by age and diagnosis. RSA response distributions over time for diagnosis and age. The interaction between time, diagnosis, and age was not statistically significant. Age dichotomized based on median split for the purpose of illustrating possible relationships

Previous research in other arousal systems, namely, the HPA axis, suggests individuals with ASD do experience elevated stress to social engagement with peers [3, 6, 71, 72]. While there were no diagnostic effects for RSA response to the TSST-F across preparation, social interaction, or recovery contexts, there were notable interactions with age suggesting developmental factors may be contributing to PNS function. Specifically, the increase in RSA with older age was blunted for the ASD group relative to the TD group, despite expected positive developmental trajectories of the PNS (e.g.), [36, 73]. The lack of change in RSA as function of age in the ASD group suggests a reduced PNS response to social engagement in the older ASD youth. Such an interaction would be consistent with previous studies of HPA axis responsivity in school-aged children (8–12 years) with ASD, which show that older children with ASD have significantly elevated stress responses to social play relative to younger children with ASD and same-aged TD peers [3, 4]. Figure 5 suggests similar trends are observed in RSA, with older ASD youth demonstrating less PNS regulation to social interaction compared to younger children with ASD and same-aged TD peers. While there was not sufficient evidence for a significant three-way, nonlinear interaction, effect size fell in the small to medium range, and follow-up studies with larger samples are necessary. While it is possible that differences may arise from an inherent atypicality in the physical development of the ANS as individuals with ASD age, there is more likely an alternative explanation, such as previous social experiences (e.g., bullying) [74, 75]; or increased insight into social difficulties [76], which shape future social anxiety (e.g.), [77] and contribute to these age effects in ASD.

The TSST-F was designed to be a relatively benign engagement protocol, meant to emulate a naturalistic face-to-face conversation with another peer. In the context of the Polyvagal Theory and Social Engagement System [13, 14, 17], this non-stressful situation should be associated with calming physiological responses and inhibition of mobilization behaviors, which in turn would promote behaviors associated with social engagement. Those who do not demonstrate the expected increase in vagal tone may be in a more mobilized state favoring hyperarousal which inhibits social engagement. Regarding the PNS, the severity of social problems was related to parasympathetic regulation, regardless of diagnostic status. Specifically, increased number of parent-reported social problems was associated with lower RSA. These findings are consistent with previous literature, such that RSA has frequently been associated with impairments in social functioning (e.g., [78]). In youth with ASD especially, lower baseline RSA and blunted parasympathetic increase to social interaction have been related to the severity of social symptoms [24, 26, 29, 30, 79].

Variability and flexibility of these arousal systems is necessary for maintaining dynamic, adaptive relationships with the environment [16, 80, 81]. Therefore, decreased variability, which is reflective of limited adaptability, is often associated with pathological conditions and may represent a state of persistent vigilance or preparation for threat mobilization [81]. While youth with ASD reported more anxiety following the interaction, self-reported state anxiety did not predict any of the physiological responses to the task. These findings are consistent with other recent work investigating perceived anxiety to social interaction [55]. It is important to note that the lack of an association between physiological arousal and perceived anxiety suggests distinct systems*.* Despite the lack of an association, it must be underscored that anxiety symptoms are prevalent in ASD, estimated to affect between 20 and 80% [82–84]. Moreover, chronic, atypical physiological arousal has been frequently cited in a number of anxiety conditions (e.g.., [64, 85, 86] Therefore, heightened responsivity to benign stimuli, though maybe not immediately associated with perceived anxiety, may contribute to persistent anxious tendencies (e.g., trait anxiety) and the development of anxiety conditions, especially as youth with ASD age.

### Limitations and future directions

Despite the rigorous approach and compelling findings across both branches of the ANS, the current study has limitations. First, although the sample was comparable to many other studies in ASD, we lacked sufficient power to examine higher order interactions, such as three-way interactions with diagnosis, social functioning, and physiology, which may have further elucidated biobehavioral profiles in youth with and without ASD. Second, social symptoms were solely measured via parent-report questionnaire reflecting general functioning whereas previous studies in other arousal systems (HPA axis) have examined observable social behavior during the interaction [3, 4, 6]. Expanded studies should similarly integrate behavioral observation in order to more precisely identify whether ANS functioning is directly associated with social engagement behaviors. Additionally, the age range in the current study was limited to school-aged, preadolescent, or early-adolescent youth and only assessed age effects at a single time point. Future studies across a wider age range, which follow youth longitudinally through developmental transitions, may further demonstrate the effects of age and related factors (i.e., insight, peer experiences) on stress responsivity. Finally, the PNS and SNS systems do not operate in isolation but are interconnected. Thus, considering their interactions within individuals will likely increase insight into unique physiological responses in ASD and their relationships with social behavior beyond studying a single system examined in isolation.

## Conclusion

The current study supports a growing literature linking atypical physiological reactivity in ASD during relatively benign social situations. The results uniquely demonstrate evidence for reduced parasympathetic functioning, especially in older youth with ASD, during a naturalistic interaction with a same-aged peer. As children are confronted with frequent social encounters with peers, the implications for atypical physiological arousal to these daily occurrences are numerous. Chronic stress might increase susceptibility to a number of conditions, including gastrointestinal problems (e.g.), [87] or internalizing disorders (e.g., [88, 89]), and impaired social engagement behaviors may increase social isolation and loneliness [90, 91], thereby increasing the risk for depression or suicidality (e.g., [92]). Future research should aim to further explain the relationships between physiology and social functioning, especially through the course of development, in order to define physiological reactivity as a potential predictive marker of physical and behavioral health risk in children and adolescents with ASD.

## Supplementary Information

**Additional file 1: Supplemental Table 1.** Model Estimates of Physiological Variables Change with a Diagnosis by Time interaction and Covarying IQ. **Supplemental Table 2.** Model Estimates of Physiological Variable Change including the Age by Diagnosis interaction and Covarying IQ.

## Data Availability

The datasets used and analyzed during the current study are available from the corresponding author upon reasonable request.

## Abbreviations

ASD: Autism spectrum disorder
ANS: Autonomic nervous system
CBCL: Child Behavior Checklist
HPA: Hypothalamic pituitary adrenal
PNS: Parasympathetic nervous system
PEP: Pre-ejection period
RSA: Respiratory sinus arrhythmia
SRS: Social Responsiveness Scale
STAIC: State Trait Anxiety Inventory for Children
SNS: Sympathetic nervous system
TSST-F: Trier Social Stress Test-Friendly
TD: Typically developing
