# Supplementary material for: Evidence for decreased parasympathetic response to a novel peer interaction in older children with autism spectrum disorder: a case-control study
Source: J Neurodev Disord. 2021 Jan 9;13:6. doi: 10.1186/s11689-020-09354-x (PMC7797088; doi:10.1186/s11689-020-09354-x)
Supplement: Supplementary file 1 — Additional file 1: Supplemental Table 1. Model Estimates of Physiological Variables Change with a Diagnosis by Time interaction and Covarying IQ. Supplemental Table 2. Model Estimates of Physiological Variable Change including the Age by Diagnosis interaction and Covarying IQ. [file 11689_2020_9354_MOESM1_ESM.docx]

**Supplemental Table 1. Model Estimates of Physiological Variables Change with a Diagnosis by Time interaction and Covarying IQ.**

| Variable | **Estimate** | ***SE*** | **df** | ***t*** | ***p*** | **(95% CI)** |
| --- | --- | --- | --- | --- | --- | --- |
| RSA |  |  |  |  |  |  |
| Intercept | 5.85 | 1.08 | 99.41 | 5.43 | <0.001 | (3.72, 7.99) |
| Diagnosis | 0.02 | 0.20 | 127.74 | 0.10 | 0.92 | (-0.38, 0.42) |
| IQ | -0.0003 | 0.005 | 99.03 | -0.05 | 0.96 | (-0.01, 0.01) |
| Time | 0.38 | 0.07 | 394.14 | 5.54 | <0.001 | (0.24, 0.51) |
| Time^2^ | -0.08 | 0.02 | 394.09 | -4.89 | <0.001 | (-0.11, -0.05) |
| Age | 0.04 | 0.08 | 98.96 | 0.56 | 0.57 | (-0.11, 0.20) |
| Diagnosis*Time | -0.08 | 0.10 | 394.09 | -0.87 | 0.39 | (-0.27, 0.10) |
| Diagnosis*Time^2^ | 0.01 | 0.02 | 394.06 | 0.29 | 0.77 | (-0.04, 0.05) |
| PEP |  |  |  |  |  |  |
| Intercept | 74.60 | 14.26 | 86.85 | 5.23 | <0.001 | (46.26, 102.96) |
| Diagnosis | 0.80 | 2.53 | 96.27 | 0.32 | 0.75 | (-4.21, 5.81) |
| IQ | -0.04 | 0.07 | 86.67 | -0.53 | 0.59 | (-0.17, 0.10) |
| Time | 0.18 | 0.55 | 346.41 | 0.34 | 0.74 | (-0.90, 1.27) |
| Time^2^ | -0.10 | 0.13 | 345.87 | -0.53 | 0.60 | (-0.33, 0.19) |
| Age | 1.65 | 1.02 | 86.75 | 1.62 | 0.11 | (-0.38, 3.68) |
| Diagnosis*Time | 0.31 | 0.79 | 346.12 | 0.40 | 0.69 | (-1.24, 1.86) |
| Diagnosis*Time^2^ | -0.13 | 0.19 | 345.71 | -0.68 | 0.50 | (-0.50, 0.24) |

**Supplemental Table 2. Model Estimates of Physiological Variable Change including the Age by Diagnosis interaction and Covarying IQ.**

| Variable | **Estimate** | ***SE*** | **df** | ***t*** | ***p*** | **(95% CI)** |
| --- | --- | --- | --- | --- | --- | --- |
| RSA |  |  |  |  |  |  |
| Intercept | 3.82 | 1.38 | 99.10 | 2.77 | 0.007 | (1.08, 6.55) |
| Diagnosis | 3.94 | 1.73 | 98.95 | 2.27 | 0.02 | (0.50, 7.38) |
| IQ | 0.0003 | 0.005 | 99.03 | 0.07 | 0.94 | (-0.01, 0.01) |
| Time | 0.34 | 0.05 | 394.09 | 6.97 | <0.001 | (0.24, 0.43) |
| Time^2^ | -0.08 | 0.01 | 394.06 | -6.63 | <0.001 | (-0.10, -0.05) |
| Age | 0.22 | 0.11 | 98.95 | 2.08 | 0.04 | (0.01, 0.43) |
| Diagnosis*Age | -0.35 | 0.15 | 98.95 | -2.34 | 0.02 | (-0.65, -0.05) |
| PEP |  |  |  |  |  |  |
| Intercept | 65.19 | 18.13 | 85.60 | 3.59 | 0.001 | (29.14, 101.23) |
| Diagnosis | 20.58 | 23.60 | 85.73 | 0.87 | 0.39 | (-26.33, 67.50) |
| IQ | -0.03 | 0.07 | 85.64 | -0.51 | 0.61 | (-0.17, 0.10) |
| Time | 0.34 | 0.39 | 348.10 | 0.85 | 0.39 | (-0.44, 1.11) |
| Time^2^ | -0.13 | 0.09 | 347.69 | -1.40 | 0.16 | (-0.32, 0.05) |
| Age | 2.47 | 1.40 | 85.53 | 1.76 | 0.08 | (-0.32, 5.26) |
| Diagnosis*Age | -1.74 | 2.05 | 85.75 | -0.85 | 0.40 | (-5.81, 2.33) |
